# Sunlight-Exposed Biofilm Microbial Communities Are Naturally Resistant to Chernobyl Ionizing-Radiation Levels

**DOI:** 10.1371/journal.pone.0021764

**Published:** 2011-07-13

**Authors:** Marie Ragon, Gwendal Restoux, David Moreira, Anders Pape Møller, Purificación López-García

**Affiliations:** Unité d'Ecologie, Systématique et Evolution - CNRS UMR8079, Université Paris-Sud, Orsay, France; New York State Health Department and University at Albany, United States of America

## Abstract

**Background:**

The Chernobyl accident represents a long-term experiment on the effects of exposure to ionizing radiation at the ecosystem level. Though studies of these effects on plants and animals are abundant, the study of how Chernobyl radiation levels affect prokaryotic and eukaryotic microbial communities is practically non-existent, except for a few reports on human pathogens or soil microorganisms. Environments enduring extreme desiccation and UV radiation, such as sunlight exposed biofilms could in principle select for organisms highly resistant to ionizing radiation as well.

**Methodology/Principal Findings:**

To test this hypothesis, we explored the diversity of microorganisms belonging to the three domains of life by cultivation-independent approaches in biofilms developing on concrete walls or pillars in the Chernobyl area exposed to different levels of radiation, and we compared them with a similar biofilm from a non-irradiated site in Northern Ireland. Actinobacteria, Alphaproteobacteria, Bacteroidetes, Acidobacteria and Deinococcales were the most consistently detected bacterial groups, whereas green algae (Chlorophyta) and ascomycete fungi (Ascomycota) dominated within the eukaryotes. Close relatives to the most radio-resistant organisms known, including *Rubrobacter* species, Deinococcales and melanized ascomycete fungi were always detected. The diversity of bacteria and eukaryotes found in the most highly irradiated samples was comparable to that of less irradiated Chernobyl sites and Northern Ireland. However, the study of mutation frequencies in non-coding ITS regions versus SSU rRNA genes in members of a same actinobacterial operational taxonomic unit (OTU) present in Chernobyl samples and Northern Ireland showed a positive correlation between increased radiation and mutation rates.

**Conclusions/Significance:**

Our results show that biofilm microbial communities in the most irradiated samples are comparable to non-irradiated samples in terms of general diversity patterns, despite increased mutation levels at the single-OTU level. Therefore, biofilm communities growing in sunlight exposed substrates are capable of coping with increased mutation rates and appear pre-adapted to levels of ionizing radiation in Chernobyl due to their natural adaptation to periodical desiccation and ambient UV radiation.

## Introduction

The explosion of one of the four nuclear reactors of the Chernobyl nuclear power plant on 26 April 1986 released huge amounts of radioactive elements into the atmosphere that polluted vast areas in adjacent continents. This catastrophic accident initiated involuntarily the largest-scale experiment to date about the effects of ionizing radiation on natural ecosystems. A wealth of studies on the consequences of radiation in Chernobyl have accumulated for more than two decades, revealing associations between levels of radiation and the abundance, distribution, life history and mutation rates of plants and animals (for review see [1], [2], [3]). Much less is known, however, about the radiation effects in eukaryotic and prokaryotic members of the microbial communities.

Among eukaryotes, fungi, both mushroom-producing species but also micro-fungi have been relatively well studied in Chernobyl [2], [3], mostly because of their capacity to concentrate radionuclides [4]. This capacity converts them into important agents for radionuclide transfer in two ways. They concentrate and mobilize radionuclides towards organisms at higher trophic levels including humans, e.g. through grazing [5]. They can also regulate radionuclide absorption. For instance, certain mycorrhiza accumulate cesium isotopes preferentially leading to their accumulation in hyphae and thus limiting their transfer to the host plant, though they may behave differently depending on the isotope and the element under consideration [5]. In addition, several micro-fungi from irradiated areas are not only radiotolerant but positively attracted by radionuclides (positive radiotropism), being able to grow upon “hot particles” and degrade them [6]. Furthermore, in some cases ionizing radiation has a positive stimulation on spore germination [7]. By contrast, positive radiotropism and stimulation of spore germination by ionizing radiation are not observed in fungi isolated from radioactively clean locations [5]. Fungal resistance to radiation is usually attributed to their smaller genomes as compared to animal or plant cells. It is also known that gamma irradiation enhances the expression of genes involved in cell cycle and DNA processing, cell rescue defense and virulence, protein and cell fate, and metabolism, as shown by microarray studies in yeast [8]. However, these factors alone do not explain the extreme radioresistance of some fungi. A key role is played by melanin and related pigments, which are invariably present in the most radiotolerant fungi. Thus, melanized fungal species, most often belonging to the Ascomycetes, colonize the walls of the highly radioactive damaged reactor at Chernobyl [9], and the relative abundance of pigmented fungi has increased in soils of the Chernobyl area [10]. The protective role of melanin is related to its physical shielding properties combined with the ability of quenching cytotoxic free radicals [11]. Furthermore, the exposure to ionizing radiation and other forms of electromagnetic radiation has been shown to increase both the growth of some melanized fungi and the electron transfer properties of melanin. This has led to speculations that these redox properties might even be used to transduce energy for cell metabolism, thereby enhancing growth [11], [12].

The study of how radiation affects other microbial eukaryotes is practically non-existent, despite the attention created by micro-fungi in Chernobyl soils and nuclear power plant reactor ruins due to their radioresistant properties. The situation is not much better for prokaryotes. There are a number of studies related to the increase of pathogenic bacteria and viruses, including retroviruses, mostly in relation to human health [13]. However, in these cases a direct radiation effect on the stimulation of, for instance, retroviral activation, cannot be easily disentangled from indirect effects due to impaired immune responses of hosts. One recent study explored the effect of Chernobyl radiation levels on cultivable bacteria and fungi from barn swallow *Hirundo rustica* feathers, which showed a negative effect of radiation on the abundance of cultivable bacteria but not of fungi [14]. However, cultivation approaches are known to yield a very biased view of the existing diversity [15], and changes induced in host metabolism, immune system and health, could indirectly influence the observed changes in the abundance of cultivable bacteria. There are relatively few studies, most of them in soils, about the effects of ionizing radiation on free-living bacteria in the Chernobyl area. These report either a decrease of bacterial diversity in highly irradiated soils or the isolation of resistant bacteria, frequently spore-forming bacilli, which are also resistant to ultraviolet (UV) radiation and H_2_O_2_ exposure [16], [17], [18], [19].

In addition to sources related to the use of nuclear power plants, research centers and hospitals, ionizing radiation occurs naturally in certain deep-sea hydrothermal vents, and high levels of radioactivity have been measured in their associated biota [20]. These conditions select for organisms that are naturally resistant to high doses of ionizing radiation (up to 30 kGy), such as *Thermococcus gammatolerans* and other highly radioresistant archaea that have been isolated from the Guaymas basin in the Pacific and the Mid-Atlantic Ridge [21], [22]. Extreme desiccation and UV radiation can also select for organisms highly resistant to ionizing radiation. This is not surprising, given that both can lead to similar effects on cellular macromolecules. UV radiation causes a variety of photochemical damages on DNA that, ultimately, may lead to mutations and to single or double strand breaks. These include, notably, the dimerization of adjacent pyrimidine bases, but also other lesions mainly derived from, specifically, UVA-derived oxidative damage [23], [24]. Similarly, ionizing radiation produces oxidative damage in DNA and proteins due to the generation of free radicals [25]. The most radioresistant organisms known to date are the bacteria *Deinococcus radiodurans* (Thermus/Deinococcus group) [26] and *Rubrobacter radiotolerans* (Actinobacteria) [27]. *Deinococcus* and *Rubrobacter* species are frequently retrieved from rocks and soils of cold and hot deserts (e.g. [28], [29], [30] or at high altitudes where the protective effect of the atmosphere against UV is diminished [31]. Several species of these genera are resistant to other extreme conditions as well, being thermophilic or resisting alkaline conditions and solvents toxic for many other organisms. Thus, some *Rubrobacter* strains have been isolated from hot springs [32] and wall paintings, where they are responsible for rosy discoloration [33]. Many isolated strains are also resistant to ionizing radiation [32], [34], suggesting that organisms adapted to xerophily and UV-radiation are naturally adapted to cope with ionizing radiation as well.

These bacteria use a variety of adaptations. Often, they protect themselves by forming endolithic communities and preventing in this way excessive UV exposure and evaporation in deserts (e.g. [35], [36], [37], but the most characteristic adaptations involve efficient DNA repair, pigments and protection from oxidative stress. *D. radiodurans* and *R. radiotolerans* possess extremely effective DNA repair systems [38], [39], [40], [41]. Furthermore, basal DNA repair genes in radioresistant bacteria of the genera *Deinococcus* and *Rubrobacter* evolve under positive selection as compared to their homologs in non-resistant bacteria [42]. The presence of carotenoid pigments is also a constant in radioresistant bacteria [43]. As in the case of melanin in fungi, which provides some fungi resistance levels nearly comparable to those of *Rubrobacter* and *Deinococcus* [11], carotenoid pigments in bacteria also provide a physical shield as well as protection from oxidative stress [44]. In addition to pigments, desiccation- and radiation-resistant bacteria possess mechanisms that limit protein oxidative damage during dehydration, which involve enzymatic and non-enzymatic antioxidant defense systems dominated by divalent manganese complexes [41], [45], [46].

In this study, we assessed the effect of ionizing radiation on communities of microorganisms that might be potentially pre-adapted to it by their natural exposure to sunlight and limited water availability. To this end, we explored the diversity of microorganisms belonging to the three domains of life by cultivation-independent approaches in biofilms developing on concrete walls or pillars in the Chernobyl area exposed to different levels of radiation, and we compared them with a similar concrete biofilm sample from a non-exposed site in Ireland. Our results show that the composition of biofilm microbial communities in the most highly irradiated samples is similar to those of non-irradiated samples, despite increased mutation levels within a same operational taxonomic unit (OTU). Therefore, biofilm communities growing in sunlight exposed substrates appear pre-adapted to ionizing radiation levels at Chernobyl due to their natural adaptation to ambient UV radiation.

## Methods

### Sample selection and background radiation levels

**Figure 1:**
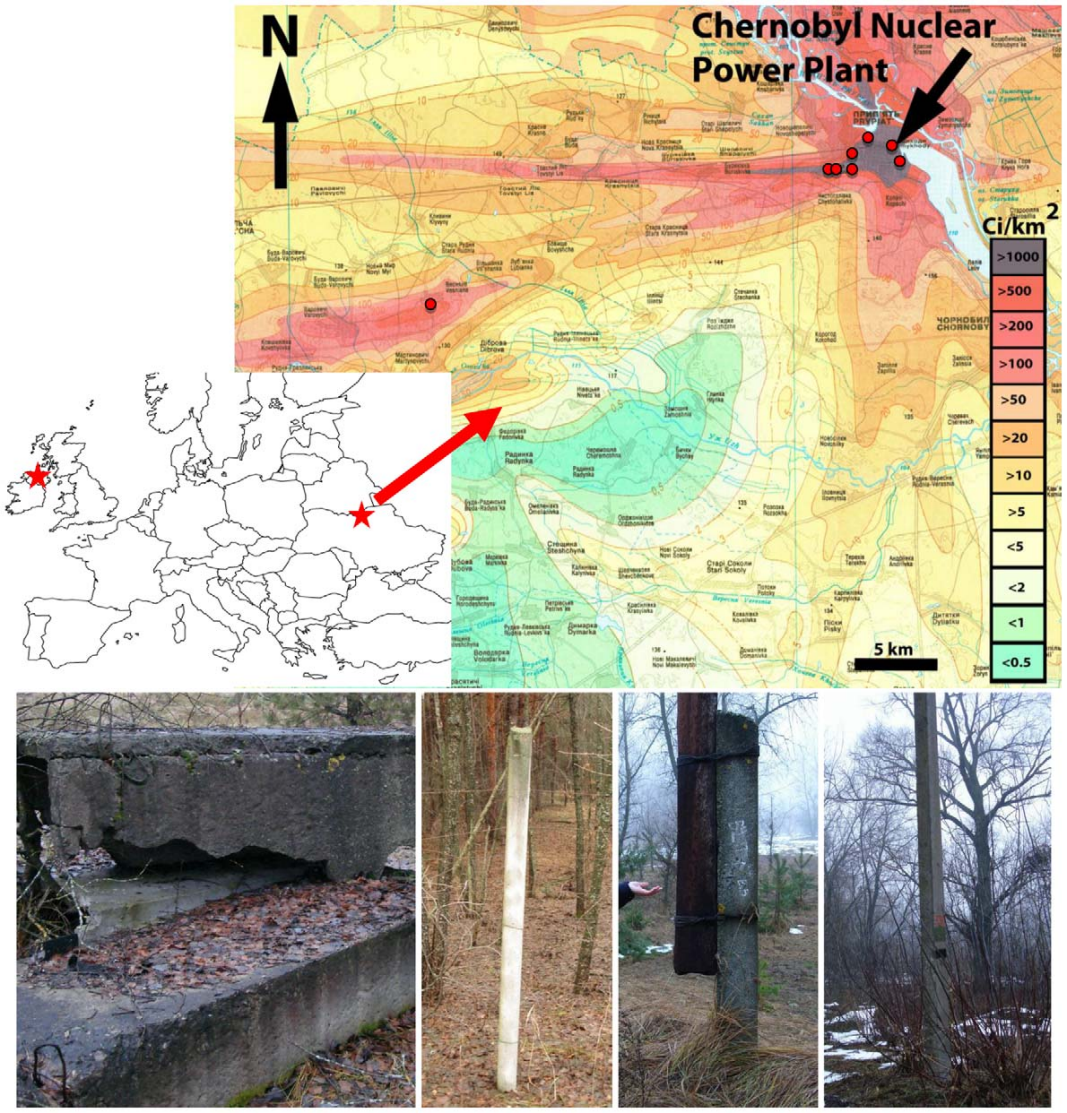
Sampling sites. The red stars indicate the two major sampling areas in Northern Ireland and Chernobyl (Ukraine). The map on the right shows the location of the sampling sites in the Chernobyl area according to the background level of radiation (µSv/h) (adapted from [47]). The photographs at the bottom show some examples of biofilms growing on concrete walls or pillars that were sampled.

We collected samples of biofilms growing on concrete pillar or wall structures in eight different points from the Chernobyl area close to Prypiat and Vesniane on December 7th, 2007 (Table 1 and Figure 1). Biofilms were dark green to black in color (Figure 1). Chernobyl samples were collected from sites with very different background levels of gamma radiation, ranging from 0.35 to 25 µSv/h, as measured *in situ* (Table 1). Gamma radiation levels in the field were measured at collection points using a hand-held dosimeter (Model: Inspector, SE International, Inc., Summertown, TN,USA) and the measurements cross-validated with Ukrainian governmental measurements [47]. In addition, we analyzed a similar concrete-associated biofilm sample from a distant geographical location (Ballyclare, Northern Ireland) but comparable latitude, which was considered as an external control in subsequent studies. The sample was collected from a concrete wall on February 19th, 2008. For this sampling point, we made an estimation of background radioactivity level of 0.08 µS/h based on the average levels from three close monitoring stations in Ireland in 2008 (Dundalk, Clones, Malinhead, with 0.073, 0.078 and 0.105 µSv/h, respectively) [48], which coincided with the long-term background radiation levels for the same sites (http://www.rpii.ie/Monitoring-Stations.aspx). This value is approximately five-fold lower than the least irradiated site sampled in Chernobyl and 300-fold less than the most irradiated one (Table 1). Ireland was also affected by the Chernobyl accident, and the initial ^137^Cs-^134^Cs deposition reached concentrations 20-fold higher than pre-catastrophe levels [49], although this represented much lower quantities than those received by most European countries [50]. In past years, the average background radiation levels in Ireland oscillated between 0.05 and 0.12 µSv/h. In all cases, biofilms were sampled by scratching the concrete surface with an ethanol-inflamed spatula and letting the detached biofilm-covered grains to fall in a sterile tube. Tubes were immediately closed and, once transported to the laboratory, stored at −20°C until further processing.

**Table 1:** Location, measured ambient radioactivity at sampling sites, number of sequences analyzed per gene library and Operational Taxonomic Units (OTUs) detected for samples analyzed in this study.

Sample name	Geographic origin	Coordinates	Radiation (µSv/h)**	Bacteria SSU rDNA sequences analyzed	Eukaryote SSU rDNA sequences analyzed	Number of bacteria OTUs	Number of eukaryote OTUs
**B1.6**	Ballyclare, Northern Ireland	54.751, −5.999	0.08*	119	130	20	6
**Cher1**	Pripyat, Chernobyl	51.23.321, 30.06.746	0.83	-	-	-	-
**Cher2**	Pripyat, Chernobyl	51.23.118, 30.05.943	8.65	70	94	9	8
**Cher3**	Pripyat, Chernobyl	51.23.462, 030.03.775	0.97	-	-	-	-
**Cher4**	Pripyat, Chernobyl (red forest)	51.23.052, 030.02.433	25	137	105	23	15
**Cher5**	Pripyat, Chernobyl	51.21.561, 030.00.595	0.35	-	-	-	-
**Cher6**	Pripyat, Chernobyl	51.21.701, 030.00.402	0.76	191	112	24	4
**Cher7**	Pripyat, Chernobyl	51.17.647, 030.38.568	6.4	-	-	-	-
**Cher8**	Vesniane, Chernobyl	51.18.380, 029.38.820	4	64	110	2	5

*This value corresponds to the average radiation levels from three close monitoring stations in Ireland in 2008 (Dundalk, Clones, Malinhead) [48].

**1 Sievert = 1 Gray.

### DNA purification, PCR amplification of SSU rDNA and ITS regions, library construction and sequence analysis

DNA extraction was done from ca. 250 µl of scratched concrete powder using the Power Soil DNA extraction kit from MoBio (Carlsbad, CA, USA) following the instructions of the manufacturer. DNA was eluted in a final volume of 80 µl 10 mM Tris-HCl, pH 8, and conserved at −20°C. Small subunit (SSU) rRNA genes were amplified using specific primers for each domain of life. In the case of bacteria, we additionally amplified the adjacent internal transcribed spacer (ITS) region, using the primers 27F (AGAGTTTGATCCTGGCTCAG) and 23S-1R (GGGTTTCCCCATTCGGAAATC). The eukaryotic SSU rDNA was amplified using the specific primers 82F (GAAACTGCGAATGGCTC) and 1520R (CYGCAGGTTCACCTAC). Finally, in order to maximize the chances to amplify archaeal SSU rRNA genes, we used combinations of the following archaea specific primers with the reverse primer 1492R (GGTTACCTTGTTACGACTT): Ar109 (AC(G/T)GCTGCTCAGTAACACGT), ANMEF (GGCTCAGTAACACGTGGA), W36 (TCCAGGCCCTACGGGG), and 21F (TTCCGGTTGATCCTGCCGGA). PCR reactions were carried out in 25 µl of reaction buffer containing 1 µl of the eluted DNA, 1.5 mM MgCl_2_, dNTPs (10 nmol each), 20 pmol of each primer, and 0.2 U Taq Platinum DNA Polymerase (Invitrogen). PCR reactions were performed under the following conditions: 35 cycles (denaturation at 94°C for 15 s, annealing at 50 to 55°C for 30 s, extension at 72°C for 2 min) preceded by 2 min denaturation at 94°C, and followed by 7 min extension at 72°C. Clone libraries were constructed using the TopoTA cloning kit (Invitrogen, Carlsbad, CA, USA) according to the manufacturer's instructions. Clone inserts were PCR-amplified using flanking vector primers, and SSU rDNA partially sequenced with either 1492R or 1520R. Accumulation curves for high-quality partial sequences were constructed using DOTUR [51] at the level of operational taxonomic units (OTUs). OTUs were defined as groups of sequences sharing more than 97% identity at the SSU rRNA gene. SSU rDNA representative of different OTUs were nearly fully sequenced by using forward primers. The ITS regions of members of a same OTU closely related to *Rubrobacter radiotolerans* present in different samples were also sequenced completely. Complete sequences were assembled using Code Aligner (CodonCode Corporation; www.codoncode.com) prior to phylogenetic analyses.

### Denaturing Gel Gradient Electrophoresis (DGGE) analysis

Fingerprinting analysis of bacterial and eukaryotic diversity in different samples was carried out by DGGE. For this, short fragments of SSU rDNA were amplified, in bacteria, using the forward GC-clamp-containing primer 341F-GC (CGCCCGCCGCGCGCGGCGGGCGGGGCGGGGGCACGGGGGGCCTACGGGAGGCAGCAG), and the reverse primer 534R (ATTACCGCGGCTGCTGG) and, in eukaryotes, the forward GC-clamp-containing primer 1209F-GC (CGCGCGCCGCGCCCCGCGCCCGTCCCGCCGCCCCCGCCCGCAGGTCTGTGATGCCC), and the reverse primer 1520R. PCR was carried out using the same reaction mix as above, except for a final concentration of MgCl_2_ of 3 mM for bacteria, and the following conditions: initial denaturation step at 94°C for 3 min, 20 cycles consisting of a denaturation step at 94°C for 15 s, an annealing step of 30 s (a touch down procedure with a decreasing annealing temperature from 65°C to 55°C for the 10 first cycles was applied followed by a hybridization temperature of 55°C for the following 10 cycles) and a polymerization step at 72°C for 1.5 min, and a final step of 7 min (eukaryotes) to 1 h (bacteria) extension at 72°C as recommended in [52]. Migration of PCR products was done in a DGGE-2000 system (CBS Scientific Company) running in parallel 50 bp ladder DNA markers (Promega, Lyon, France). An 8% polyacrylamide gel with a gradient of DNA denaturant agent was cast by mixing solutions of 30% to 60% for bacteria, and 20% to 40% for eukaryotes. 100% denaturant agent is 7 M urea and 40% formamide. 10–15 µl of PCR product were loaded for each sample and the gel was run at 150 V for 6 hours at 60°C in 0.5× Tris-acetate-EDTA (TAE) buffer (1×: 40 mM Tris, 20 mM acetic acid, 1 mM EDTA, pH 8.0). After electrophoresis, gels were stained in SYBR Gold (Molecular Probes, Invitrogen, USA), and then visualized and photographed on a UV transilluminator (TFX-26.LM, VWR International, France). Photographed gels well normalized using the Bionumerics® 5 software (AppliedMaths, Saint-Martens-Latem, Belgium) based on the position of ladder standards. Band positions were assigned manually. Clustering analysis of DGGE patterns was carried out using UPGMA (Unweighted Pair Group Method with Arithmetic mean) clustering [53] applying a Jaccard coefficient [54].

### Phylogenetic analyses

Our sequences were compared to those in GenBank by BLAST [55]. We retrieved the closest sequences identified in this way to include them in an alignment containing also sequences from the closest cultivated members and some representative sequences of the major taxa found. Sequences were aligned using MUSCLE [56] and the multiple alignment was then manually edited using the program ED from the MUST package [57]. Preliminary neighbor-joining (NJ) trees were constructed for the different prokaryotic taxa in order to choose representative subsets of sequences for further phylogenetic analyses. Finally, phylogenetic trees were then reconstructed using final datasets by maximum likelihood (ML) using TREEFINDER [58] applying a general time reversible (GTR) model of sequence evolution, and taking among-site rate variation into account by using a four-category discrete approximation of a Γ distribution. Gaps and ambiguously aligned positions were excluded from our analysis. ML bootstrap proportions were inferred using 1000 replicates. Phylogenetic trees were viewed using the program FIGTREE (http://tree.bio.ed.ac.uk/software/figtree/). The sequences reported in this study have been deposited in GenBank with accession numbers JN020169-JN020245.

### Modeling the effect of ionizing radiation on genetic diversity at a single OTU level

To model radiation effects on the genetic variability at intra-species (microdiversity) level, we used as samples 36, 20 and 30 SSU rDNA plus ITS sequences belonging to the same *Rubrobacter* OTU coming from three Chernobyl locations differing by their radiation levels (Cher2, Cher6 and Cher8). We compared these sequences to three sequences (Ref-127, Ref-128 and Ref-2 corresponding to clones B1.6-1B-127, B1.6-1B-128 and B1.6-1B-2, respectively) of the same OTU retrieved from the control, non-irradiated Northern Ireland sample. Each datum was obtained by counting the number of differing nucleotides for the ITS (355 bp) and for the SSU rDNA (1387 bp) regions sequenced between each Chernobyl sample and the reference sample. We used a Generalized Linear Model (GLM) [59] to regress the observed variability at the ITS by the SSU rDNA variability (quantitative variable). This regression accounts for the control sequence used (qualitative fixed effect) and the Chernobyl population from where it was retrieved (qualitative fixed effect). We assumed that the number of differences for the ITS followed a Poisson distribution and we used a square-root link function to linearize the observed distribution. We only considered interactions up to the second order (the third order interaction term was ignored since it did not bring any additional information). The model was thus designed as follows,
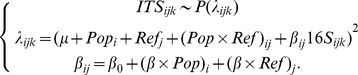
With *ITS* and *16S* being the number of different nucleotides of a given sequence against the reference sample sequence for the two DNA regions respectively, *μ* the general mean, *Pop* the population to which the sequence belongs, *Ref* the control reference sequence used for comparison, *Pop x Ref* the interaction term and *β* the regression coefficients. The effects were tested using a type III analysis by setting the contrasts to “effects sum equals zero” and applying the *drop1* function. Finally, we computed the adjusted means for each population and reference sample assuming a comparable level of SSU rDNA (*16S*) variability which was the average level of SSU rDNA variability computed over all the dataset. This enables comparing the “real” effect of populations on the genetic variability of the ITS regions for exactly the same level of SSU rDNA variation, i.e. without the confounding effect of SSU rDNA variability. All computations were done using R statistical language R [60] and the “Effects” package [61].

## Results and Discussion

### Prokaryotic diversity of Chernobyl concrete biofilm communities

To carry out in-depth analyses of the microbial diversity associated with the sunlight-exposed biofilms growing on concrete, we made a sub-selection of samples from Chernobyl that covered the whole range of radiation measured plus an outgroup sample from Northern Ireland (Figure 1 and Table 1). First, we aimed at studying the diversity of the two prokaryotic domains, Archaea and Bacteria, in biofilms by amplifying SSU rRNA genes using specific-domain primers. However, in the case of archaea and despite the use of several primer combinations under conditions that are known to allow the amplification of this marker, we failed to obtain amplicons. Since archaea are known to be much less relatively abundant than bacteria in most habitats [62], we also applied nested PCR to maximize the probability of amplifying archaeal SSU rDNA, with identical negative results. This strongly suggests that archaea are not present in this type of biofilms, or, if they occur, they do in far too small amounts to be detectable by molecular methods. This result is in agreement with additional observations in sunlight exposed biofilms on other mineral substrates (Ragon et al., unpublished). Archaea have very rarely been reported to occur on monument surfaces, and when they do in certain particular samples, their diversity corresponds to that of halophilic archaea, which can cope more easily with the higher osmotic stress often associated to these systems [63], [64], [65]. Haloalkaliphilic archaea are frequent in paintings, which is likely due to their adaptation to high salt (including metal cation) concentrations and organic solvents [66] [33].

**Figure 2:**
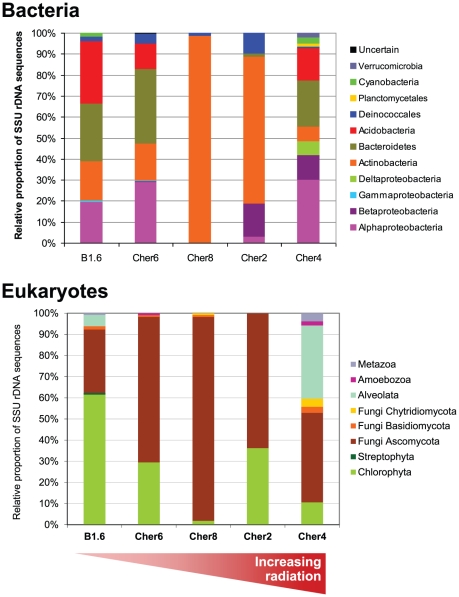
Relative distribution of major bacterial and eukaryotic taxa in SSU rRNA gene libraries from concrete-associated biofilm microbial communities.

Bacterial SSU rRNA genes were easily amplified together with the adjacent ITS region, and subsequently cloned. The total number of sequences analyzed per sample is given in Table 1. In total, we analyzed nearly six hundred bacterial sequences. Some samples reached saturation rapidly, and therefore, the number of sequences sampled is smaller for the corresponding SSU rDNA libraries. That was in particular the case for Cher8, but also for Cher2 and Cher6. Contrary to our expectations, the sample from the most highly irradiated site, Cher4, was the only one for which saturation was not clearly reached, whereas the sample from Northern Ireland seemed to reach an asymptote, suggesting that Cher4 harbored a higher bacterial diversity (Figure S1). Cher4 diversity was not only higher in terms of OTUs present, but also in terms of higher-order taxa, as deduced from BLAST comparisons with sequences deposited in GenBank and phylogenetic analyses. The relative distribution of sequences in high-rank taxonomic taxa from the most irradiated site, Cher4, resembled more the less radiation-exposed sites, the control Northern Ireland sample (B1.6) and Cher6, than the other Chernobyl sites (Figure 2). These samples shared relative similar proportions of dominant phyla, with Alphaproteobacteria comprising between ca. 20–30% SSU rDNAs in gene libraries, Bacteroidetes between ca. 20–35%, Acidobacteria between ca. 15–30% and Actinobacteria around 20% in B1.6 and Cher6. Actinobacteria were slightly less abundant (ca. 10%) in Cher4, where Betaproteobacteria represented ca. 12% of sequences in gene libraries. By contrast, Cher8, a sample of intermediate background radiation, was very poor in terms of bacterial diversity with 98% Actinobacteria and the remaining 2% corresponding to Deinococcales. The sample Cher2, the second most highly irradiated site had an intermediate profile between Cher8 and the rest, with a highly dominant proportion of Actinobacteria (ca. 70%), 17% of Betaproteobacteria and and 10% of Deinococcales, but it also contained Alphaproteobacteria and Bacteroidetes, though in much lower proportions than the rest of samples except Cher8 (Figure 2). Finally, cyanobacterial sequences were only detected, though in relatively low amounts, in the Northern Ireland (B1.6) and Red Forest (Cher4) samples which, being at both extremes of the background radiation levels, showed the highest diversity.

**Figure 3:**
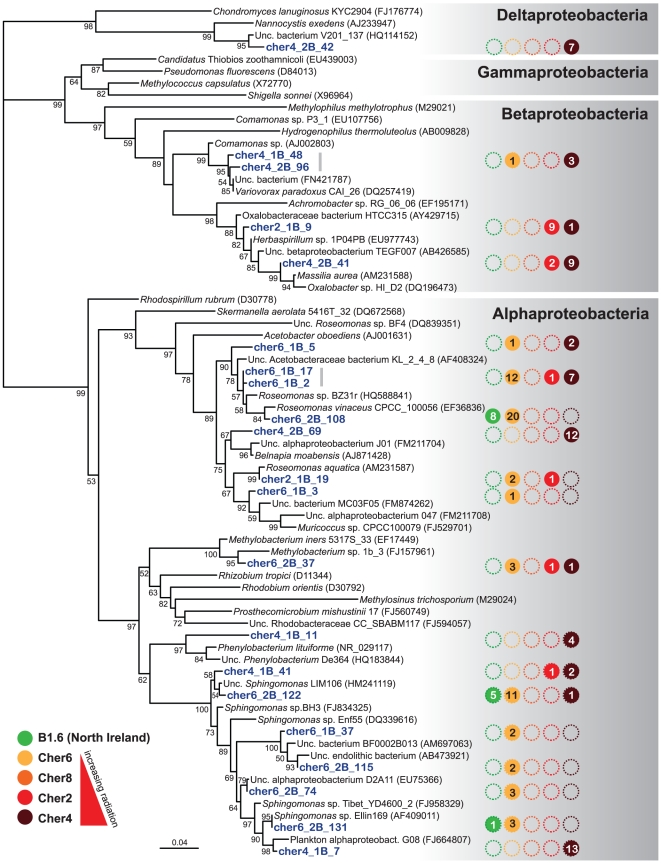
Phylogenetic tree of SSU rDNA sequences from Chernobyl concrete biofilms belonging to the Proteobacteria. The tree was reconstructed by maximum likelihood using 767 non-ambiguously aligned positions. Bootstrap values higher than 50% are given at nodes. Colored circles show the presence of the OTU in the different samples, and the internal number corresponds to the number of occurrences of sequences in the corresponding gene libraries. Unc., uncultured.

The relatively low diversity of cyanobacteria and their detection in only two of the analyzed samples might seem surprising, since the presence of primary producers is a priori needed to sustain such a high diversity of typical heterotrophic bacteria phyla (Actinobacteria, Bacteroidetes, Acidobacteria, Deinococcales). Some Alphaproteobacteria are photosynthesizers, but this is most probably not the case for most phylotypes encountered in Chernobyl biofilms, since they are not closely related to typically photosynthetic genera (Figure 3). There are two, not mutually exclusive explanations for this. First, a primary production activity seems assured in these samples by eukaryotic algae, which are present in all biofilms sampled (Figure 2; see below). Second, there might also be a bias against cyanobacteria due to their thick sheaths of exopolymeric substances, which makes cell lysis more difficult as compared to other bacteria. Consequently, the amount of cyanobacterial DNA is usually lower, which translates into lower observed frequencies in gene libraries [67].

The overall bacterial diversity observed at the phylum level was paralleled by a relatively high intra-phylum or intra-class diversity in all the studied samples to the exclusion of the low-diversity sample Cher8. The most diverse group was that of the Alphaproteobacteria, with up to 14 different phylotypes or OTUs detected (Figure 3). Alphaproteobacteria constitute an extremely successful group of bacteria, encompassing a wide variety of metabolisms, from photo- and chemoautotrophy to photoheterotrophic and purely heterotrophic strategies [68]. In our samples, the most represented OTUs were related to various members of the genus *Roseomonas*, whose members produced pink-pigmented colonies and have been isolated from a variety of environments, including aquatic systems [68]. The closest cultivated member to any of our OTUs was the heterotrophic *R. aquatica* [69]. We also identified one phylotype branching within the C1-metabolizing bacteria of the genus *Methylobacterium*, also pink-pigmented bacteria frequently isolated from biofilms associated with the same kind of drinking-water systems and lakes [69], [70]. Together with relatives of the genus *Roseomonas*, OTUs related to the alphaproteobacterial genus *Sphingomonas* were the most abundant and diverse. Members of this heterotrophic genus are found in a variety of environments, from soils to the deep subsurface and to endolithic communities in Antarctica, being involved in the degradation of complex aromatic compounds [71], [72], [73]. Together with the Alphaproteobacteria, members of the Betaproteobacteria were also present in the two most highly irradiated samples. Like Sphingomonadales, Betaproteobacteria are frequently involved in the degradation of chloroaromatic compounds, being frequently found in polluted soils and subsurface environments [74], [75]. We also detected one deltaproteobacterial OTU related to the gliding-bacteria of the order Myxococcales in Cher4 (Figures 2 and 3).

**Figure 4:**
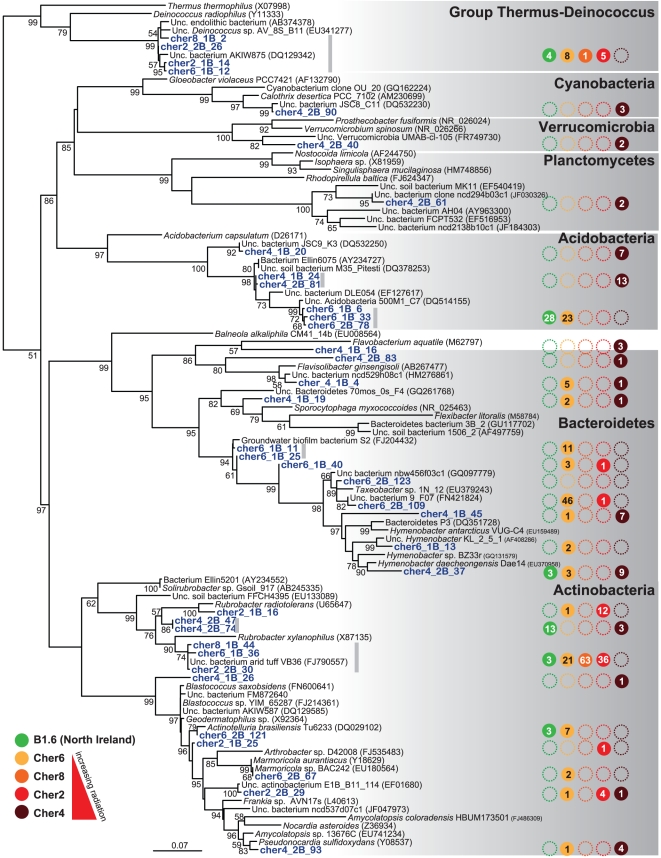
Phylogenetic tree of bacterial SSU rDNA sequences to the exclusion of Proteobacteria retrieved from sunlight-exposed biofilms in Chernobyl. The tree was reconstructed by maximum likelihood using 634 non-ambiguously aligned positions. Bootstrap values higher than 50% are given at nodes. Colored circles show the presence of the OTU in the different samples, and the internal number corresponds to the number of occurrences of sequences in the corresponding gene libraries. Unc., uncultured.

In addition to the Alphaproteobacteria, the two most abundant and diverse groups were Actinobacteria and Bacteroidetes with, respectively, ca. 10 different OTUs detected in the different biofilm samples (Figure 4). Acidobacteria, though relatively abundant, were only represented by three OTUs, which were related to either uncultured acidobacteria from soil or soil isolates [76]. Bacteroidetes, together with members of the less represented phyla Verrucomicrobia and Planctomycetales, are also frequently found in soils and sediments, being fundamentally heterotrophic bacteria. Within the Gram positive Actinobacteria, several OTUs were related to uncultured microorganisms but also to known genera, such as *Marmoricola* or the pigmented *Geodermatophilus*. However, by far, the most represented OTUs were related to the genus *Rubrobacter*, and more particularly, to *R. radiotolerans*, the most radiotolerant bacterium known to date [32], [77]. In particular, the OTU represented by sequences cher2_2B_30, cher8_1B_44 and cher6_1B_36 (Figure 4) was dominant in most Chernobyl samples, though not detected in the most irradiated site, Cher4, where other *Rubrobacter*-related OTUs were detected. This OTU was also present in the control Northern Ireland sample. It was the only phylotype detected in the low-diversity Cher8 sample together with one *Deinococcus*-related sequence (Figures 2 and 4). The desiccation- and radiation-resistant Deinococcales were systematically present in all samples, though in low numbers, and were all closely related to a *Deinococcus* sequence retrieved from an endolithic sample from Weissenstein (AB374378).

### Eukaryotic diversity of Chernobyl concrete biofilm communities

We also characterized the diversity of microbial eukaryotes associated with the sunlight exposed biofilms in Chernobyl and Northern Ireland samples by amplifying, cloning and sequencing SSU rRNA genes. The overall taxonomic distribution of sequences in gene libraries is shown in Figure 2. As in the case of bacteria, the sample from the Red Forest (Cher4) exhibited the largest variety of eukaryotic groups present, being comparable to that of the control sample from Northern Ireland. However, there was a remarkable constancy in all the samples regarding the presence of green algae (Chlorophyta) and ascomycete fungi. These two groups accounted for more than 90% (and up to 100% in the second most irradiated site, Cher2) in all the samples, except for Cher4, where they accounted only for slightly more than 50%. However, their relative proportions varied, with green algae dominating in B1.6 (ca. 60%) and ascomycetes dominating in Chernobyl samples (from 45 to 98%). In the case of Cher4, a relatively large proportion of ciliate (Alveolata) sequences were detected. Since ciliates have two nuclei, with the number of gene copies highly amplified in the macronuclei, the presence of a few cells can lead to an overrepresentation of sequences in the sample. This may be also the case if metazoans are present, since they contribute with more cells per individual and, hence, relatively larger amounts of DNA. For this reason, the relative proportions of these groups in libraries should be interpreted with caution, since they may not reflect actual organismal proportions. Both metazoans (rotifers) and ciliates are present in this sample but also, though in lower proportions, in the North Irish sample. This suggests that the communities of these biofilms are relatively rich. A few minor groups were also detected in various samples. These included a few sequences of basidiomycete fungi in several samples, but also of chytrids, which are flagellated anaerobic fungi, frequently parasitic or living in environments rich in organic matter [78], [79].

**Figure 5:**
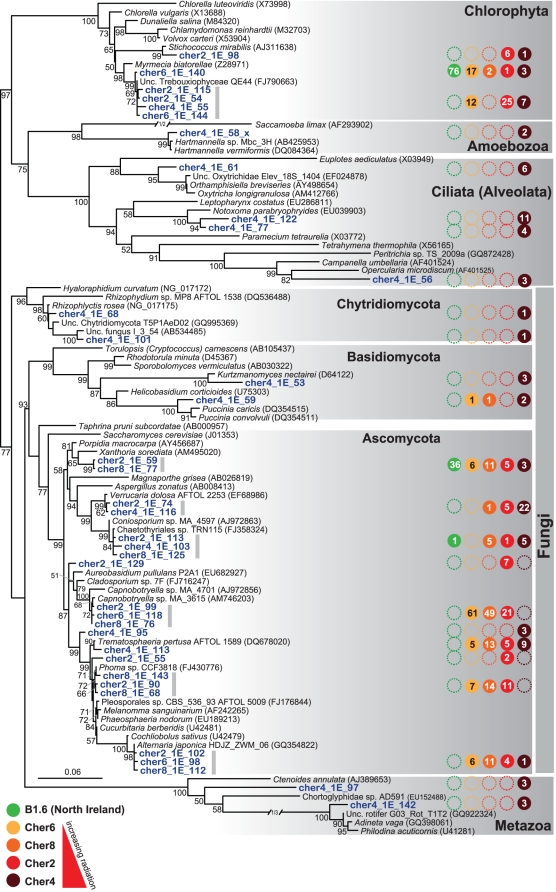
Phylogenetic tree of eukaryote SSU rDNA sequences from Chernobyl sunlight-exposed biofilms. The tree was reconstructed by maximum likelihood using 1,199 non-ambiguously aligned positions. Bootstrap values higher than 50% are given at nodes. Colored circles show the presence of the OTU in the different samples, and the internal number corresponds to the number of occurrences of sequences in the corresponding gene libraries. Unc., uncultured.

Ascomycota was not only the most abundant group, but also the most diverse, with ten different OTUs detected in the biofilm samples, many of which were present in several Chernobyl samples and some even in the control Northern Ireland sample (Figure 5). The most widespread phylotype was the one represented by sequences cher2_1E_59 and cher8_1E_77, which was closely related to sequences of the genus *Xanthoria* (98% identity to *X. parietina*). Though ubiquitous, it was more abundant in the Northern Ireland sample. *Xanthoria* species are usually found as mycobionts in lichens, most often associated with green algae of the genus *Trebouxia* [80]. The most relatively abundant phylotype in samples where it was present (Cher2, Cher6, Cher8) was related to *Capnobotryella* (representative sequences cher2_1E_99, cher6_1E_118, cher8_1E_76). *Capnobotryella* is a black fungus frequently retrieved from monument surfaces, including energy transmission towers, and windows as well as desert areas [81], [82], [83], [84]. *Capnobotryella* appears to colonize lichens in an opportunistic manner [82]. This phylotype was not found in the highly radiated sample Cher4, where another phylotype closely related to *Verrucaria* dominated (Figure 2). *Verrucaria* has been found forming endolithic desert lichens or lichens colonizing newly constructed stone structures [85], [86]. In addition to the clearly dominant ascomycetes, we detected two different phylotypes for both basidiomycetes and chytrids, but only in the most diverse sample, Cher4. This sampling point also showed a relatively large diversity of ciliates, with four different phylotypes, suggesting that ciliates do not occur accidentally in this type of samples, but that they are relatively diverse and thrive on the biofilm surface, acting as grazers. Also amoebozoan sequences belonging to the genus *Hartmanniella* were detected in Cher4. Like several of the alphaproteobacteria identified, these amoebae are frequently detected in biofilms of drinking water systems. They usually harbor endosymbiotic bacteria, mostly belonging to the Alphaproteobacteria and Chlamydiae [87], [88].

Among photosynthetic eukaryotes, although green algae constituted an abundant group in most samples (Figure 2), their diversity was rather poor, in contrast to the ascomycete fungi. Three different phylotypes were identified, two of them were closely related among them and also related to the genus *Trebouxia* (98% similarity), while a third phylotype was related to the genus *Stichococcus*, all within the Trebouxiophyceae (Figure 5). Members of the two genera are known to form lichens, acting as photobionts, and very often they are found in endolithic communities. The most abundant phylotype in most samples was closest to the environmental Trebouxiophyceae sequence QE44 (FJ790663), which was retrieved from quartz pavement in high altitude tundra of central Tibet [89]. Members of this group also form cryptoendolithic communities in Antarctica [35]. Many Trebouxiophyta, including *Trebouxia* species produce mycosporine-like aminoacids, which play an important role in the protection of these lichen-forming algae against UV radiation [90], [91]. They are also resistant to metals [92].

Given the co-existence and relative high abundances of *Trebouxia*-related phylotypes with *Xanthoria* and *Verrucaria*, it is likely that they form lichens, perhaps with some intervention of *Capnobotryella*, though both photobiont and mycobiont can exist and disperse as free-living forms [80], [93].

### Comparison of DGGE fingerprints from different biofilm communities

**Figure 6:**
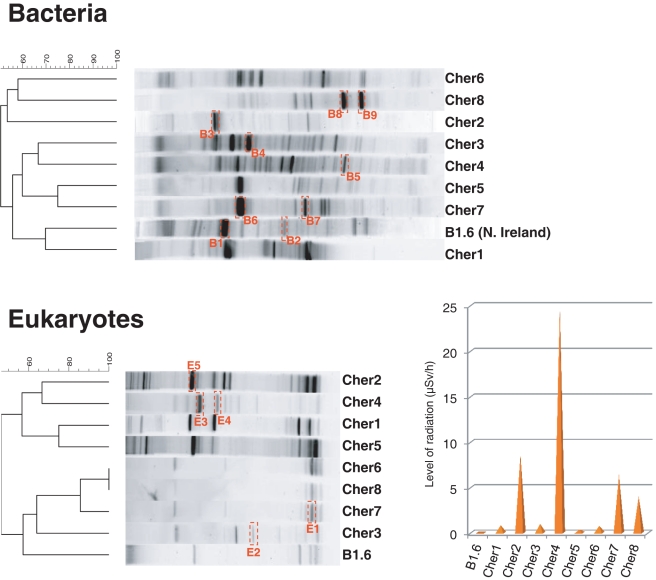
Cluster analysis of DGGE fingerprints for bacteria (A) and eukaryotes (B) in Chernobyl concrete biofilms. The histogram shows the relative levels of radioactivity measured *in situ* in Chernobyl samples (µSv/h).

We extended our comparative analysis of diversity profiles to the rest of Chernobyl samples to better cover the range of radiation levels and to limit potential effects due to local heterogeneity. For this, we carried out DGGE fingerprinting analyses for both bacteria and eukaryotes based on the amplification of a SSU rDNA fragment in all Chernobyl samples, including the samples that we analyzed in greater detail by constructing gene libraries, four additional Chernobyl samples (Table 1) plus the control B1.6 sample from Northern Ireland. We then clustered the samples as a function of the presence or absence of bands in the DGGE patterns (Figure 6). The results obtained by this approach confirmed the observation already made by analyzing the SSU rRNA gene libraries. First, the patterns observed, particularly in the case of bacteria, contained numerous bands, suggesting that bacteria are diverse in these samples. Second, a specific trend that would tend to group DGGE patterns of high radiation versus low radiation samples was not apparent. In the case of bacteria no clear grouping of patterns was visible. In the case of eukaryotes, samples Cher6, Cher7 and Cher8 showed very similar patterns with few bands, although they varied in relative intensities (Figure 6). Actually, the eukaryotic diversity between Cher6 and Cher8 as deduced from gene libraries was qualitatively similar, though in Cher8 ascomycetes proved to be much more abundant than green algae (Figure 2).

In order to facilitate the association between the DGGE patterns and the bacterial and eukaryotic diversity identified by SSU rDNA sequence analysis, we cut off some of the distinctive and more intense bands in gels, purified the DNA and sequenced the SSU rDNA fragments once re-amplified (Figure 6). As shown in Table 2, those bands corresponded to bacterial or eukaryotic lineages already identified in gene libraries. Two of the intense bands, in particular in Cher7, but also in Cher2, corresponded to cyanobacteria, suggesting that these photosynthetic bacteria are also present in samples, but underrepresented in SSU rDNA libraries. The other bacterial DGGE bands that we identified corresponded to Alpha- and Betaproteobacteria, Bacteroidetes and Actinobacteria. In the case of eukaryotes, most of the DGGE bands analyzed corresponded to ascomycete fungi, one band to a green alga and another one to a rotifer (Table 2). These data correlated well with the diversity observed from SSU rRNA gene library analysis and confirmed the quantitative importance of some phylotypes.

**Table 2:** Taxonomic affiliation of relatively abundant and distinctive bacterial and eukaryotic SSU rDNA fragments from DGGE gels (Figure 6) and correspondence with sequences from SSU rRNA gene libraries.

Band	First Hit	% identity	Phylogenetic ascription	Corresponding OTU
**BACTERIA**				
B1	cher4_2B_90	89	Bacteria; Cyanobacteria; Nostocales	cyanobacteria_OTU1
B2	cher4_2B_41	91	Bacteria; Betaproteobacteria; Burkholderiales	betaproteobacteria_OTU5
B3	cher4_2B_90	88	Bacteria; Cyanobacteria; Nostocales	cyanobacteria_OTU1
B4	cher4_2B_95	100	Bacteria; Bacteroidetes; Sphingobacteria	bacteroidetes_OTU1
B5	cher2_1B_19	94	Bacteria; Alphaproteobacteria	alphaproteobacteria_OTU5
B6	cher4_2B_90	88	Bacteria; Cyanobacteria; Nostocales	cyanobacteria_OTU1
B7	cher6_1B_26	93	Bacteria; Bacteroidetes; Sphingobacteria	bacteroidetes_OTU3
B8	cher2_1B_2	98	Bacteria; Actinobacteria	actinobacteria_OTU14
B9	cher2_1B_2	99	Bacteria; Actinobacteria	actinobacteria_OTU14
**EUKARYOTES**				
E1	cher2_1E_137	97	Fungi, Ascomycota	ascomycota_OTU5
E2	cher2_1E_115	87	Viridiplantae; Chlorophyta	chlorophyta_OTU3
E3	cher2_1E_113	95	Fungi, Ascomycota	ascomycota_OTU10
E4	cher2_1E_129	95	Fungi, Ascomycota	ascomycota_OTU7
E5	cher4_1E_142	89	Metazoa; Rotifera	metazoa_OTU2

### Mutation rates in ITS versus SSU rDNA regions in members of the same OTU from samples with different background radiation levels

**Figure 7:**
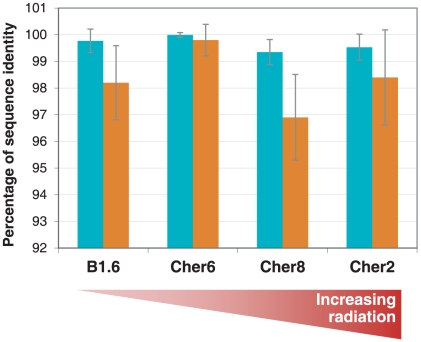
Percentage of sequence identity at SSU rRNA genes and adjacent ITS regions in the same *Rubrobacter* OTU from different populations. Note that the scale at the ordinates begins at 92% to maximize potential differences between samples and markers. The bars correspond to the standard deviation.

Increased mutation rates have been observed in animals and plants in the Chernobyl region after the accident, and this may also apply to some human-pathogenic bacteria and retroviruses [13]. However, this kind of studies is missing for natural populations of free-living bacteria. We took advantage of the fact that one of the most abundant bacterial OTUs, which is ascribed to the genus *Rubrobacter* (cher2_2B_30, cher8_1B_44 and cher6_1B_36), was present in relative high proportions (>20 sequences per sample) in three Chernobyl samples experiencing different ionizing-radiation levels and also in the control sample from Northern Ireland (3 occurrences) (Figure 4). We then sequenced the ITS region adjacent to the SSU rRNA gene for an equivalent number of clones in the Chernobyl samples (20, 30 and 36 for Cher6, Cher8 and Cher2, of increasing background radiation levels, respectively). Since the ITS is a non-coding region, it is generally considered as neutral marker when compared to highly conserved genes subject to strong purifying selection, such as the SSU rRNA gene. If ionizing radiation had a positive effect on the mutation rate of these bacteria, we would expect that a higher number of substitutions or single nucleotide polymorphisms (SNPs) accumulate in the ITS region as compared to the SSU rDNA (the OTU's identity marker within a limited range of sequence identity, usually >97%) as background radiation increases. In our case, there was some variation between populations at the SSU rDNA level, but in all cases the average percentage of nucleotide identity within and among populations was higher than 99%, which corresponds to a conservative definition of OTU for bacteria. The ITS regions were more variable, with averages varying between nearly 97% to nearly 100% nucleotide identity among populations (Figure 7). The highest difference between ITS and SSU rDNA average substitution frequency was found in the low-diversity sample Cher8, and the lowest in Cher6, where both SSU rDNA and ITS regions were remarkably homogeneous. Therefore, despite slight differences between samples in terms of average substitution rates of ITS versus SSU rDNAs, a potential correlation of these with increasing radiation was not apparent from this first-hand inspection. Notably, average SSU rDNA and ITS percentage nucleotide identities were very similar between the non-irradiated North Irish site and the most irradiated sample in this case, Cher2 (Figure 7).

**Figure 8:**
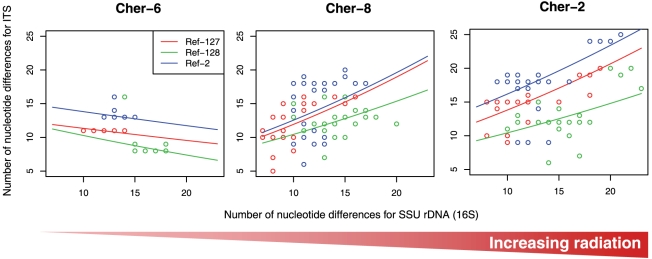
Relationship between SSU rRNA and the corresponding ITS variability (number of differing nucleotides) by comparison with Northern Ireland control sequences. Each graph corresponds to a sampled location in the Chernobyl area. Each color represents a particular reference sequence: red (Ref-127), green (Ref-128), blue (Ref-2).

Nevertheless, in order to get a deeper insight in these observations and to fully discern the effects of radiation on both genetic markers we used statistical modeling. This approach allowed us to tell apart the effects of natural phylogenetic variation within a same OTU (linked to the intrinsic SSU rDNA variability) from that of ionizing radiation acting upon a supposedly neutral marker such as the ITS. We found an overall significant and positive relationship between the variability observed for the SSU rRNA and the ITS sequences when using the Irish external reference (Table 3). First, as expected, larger differences for the SSU rRNA correlated with larger differences for the ITS region within sequences belonging to the same OTU. Interestingly, this statistical analysis revealed a significant effect of the considered population on this relationship (Table 3). The less irradiated Cher-6 population exhibited no relationship between SSU rRNA and ITS variability, whereas the samples from the Cher-8 and Cher-2 populations, subject to increasing radiation levels, exhibited positive correlations, with more phylogenetically divergent SSU rDNA sequences being associated with more divergent ITS sequences (Figure 8). The absence of positive correlation in SSU rDNA/ITS substitution frequency in Cher-6 result is due to the very weak level of ITS variation relative to the SSU rDNA. This might seem surprising since the ITS region should exhibit higher variations than the highly conserved SSU rDNA. However, the Cher-6 sample is the smallest one (20 sequences) and, consequently, a raw data analysis of this kind should be considered with caution. For instance, observed differences at the SSU rRNA among the three sampled populations could lead to misinterpretation about population or reference-sequence effects. To get rid of such potential biases, i.e. of the ITS variability due to natural intra-OTU SSU rDNA variability, we computed adjusted means of the number of expected differences for the ITS region for the same level of SSU rRNA variability that we set to the general mean over all samples (i.e. about 13 different nucleotides per sample with the references sequences). These computations were made using the estimated coefficient of the statistical model (Table S1).

**Table 3:** Summary of the tests of the different effects using a deviance analysis using Type III decomposition.

Effects	df	Deviance	F value	Pr(F)	Significance
16S	1	118.33	7.0777	0.008	**
Pop	2	117.11	2.2385	0.109	
Ref	2	115.68	0.7310	0.482	
16SxPop	2	118.33	3.5353	0.031	*
16SxRef	2	115.60	0.6406	0.528	
PopxRef	4	122.25	3.8508	0.005	**

*P<0.05,

**P<0.01.

16S (SSU rDNA) and all subsequent terms implying this variable are regressors. Pop (Chernobyl population) and Ref (North Irish reference sequence) are qualitative effects.

**Figure 9:**
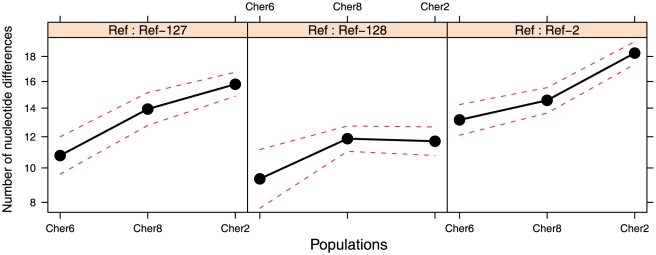
Adjusted means by population of the number of differing nucleotides as compared to the control Northern Ireland sequences (Ref-127, Ref-128, Ref-2 from left to right). The Y-axis represents the number of expected differences when assuming a similar level of SSU rRNA variation set to the general mean (i.e. ca. 13 differing nucleotides).

When the effect of intra-OTU SSU rDNA variability was removed, we observed a clear difference between populations at the level of ITS substitution frequency, with the less radiation-exposed site having significantly less ITS mutations than the more exposed sites (Figure 9). Thus, Cher-6, the less radiated population, always exhibited the lowest ITS diversity level (Figure 9) whatever the reference sequence. Moreover, when considering Ref-127 and Ref-2 as control sequences, the divergence ranking was in accordance with the exposure level, most exposed populations exhibiting the highest level of nucleotide substitutions. The results obtained from the Ref-128 control sequence showed a less clear pattern, with larger confidence intervals, although still showing a clear trend toward less divergent ITS sequences for the Cher-6 population (Figure 9). These results support the hypothesis of a larger mutation rate induced by increasing ionizing-radiation levels around Chernobyl. Furthermore, given that the ITS sequence used in this study was relatively short (355 bp) compared to the SSU rRNA fragment sequence (1387 bp), the use of longer ITS or other neutral marker sequences would probably have strengthened the observed pattern.

### Concluding remarks

In the present work, we explored the microbial diversity of microorganisms forming biofilms on concrete pillars and walls being naturally exposed to sunlight in sites with different levels of background ionizing-radiation in the Chernobyl area and compared it with that found at a geographically distant control site in Northern Ireland. We aimed at describing the microbial diversity in a comprehensive way by studying microorganisms from the three domains of life and at different resolution levels, from the relative distributions of high-rank taxa to OTU and even intra-OTU (“intraspecific”) levels. Despite intense efforts, recurrent failure to amplify archaeal SSU rRNA genes with several primer combinations strongly suggests that members of this domain of life are not present in this type of concrete-associated biofilms or are present in very minor proportions. Although some differences in the relative proportions of bacteria and microbial eukaryotes exist between the different samples, the most characteristic and/or relatively abundant taxonomic groups present in these biofilms seem to be Actinobacteria, Alphaproteobacteria, Bacteroidetes, Acidobacteria and Deinococcales within the bacteria and green algae (Chlorophyta) and ascomycete fungi (Ascomycota) within the eukaryotes.

Some members of the Actinobacteria (particularly *Rubrobacter* species), Deinococcales and melanized ascomycete fungi are the most radio-resistant organisms known, and it could be hypothesized that their presence in Chernobyl is a consequence of the selection imposed by high ionizing radiation. According to this view, a decrease in the specific diversity should also be observed with increasing radiation. However, our studies indicate that the diversity of bacteria and eukaryotes found in the most highly irradiated samples is comparable (or even higher) than that found in less irradiated Chernobyl sites and in Northern Ireland. This can be seen at different levels of resolution, from high-rank taxa diversity (Figure 2), to OTU diversity (Figures 3, 4, 5) in the samples studied by sequencing clones from SSU rRNA gene libraries, but can also be seen in additional samples from Chernobyl by DGGE fingerprinting analyses (Figure 6). This does not mean that radiation does not affect these microbial communities, since the study of mutation frequencies in non-coding ITS regions versus SSU rRNA genes in members of a same actinobacterial OTU present in samples from Chernobyl and Northern Ireland showed a positive correlation between increased radiation and mutation rates. Therefore, a comparable microbial diversity is maintained in spite of higher mutation rates in sites exposed to increasing radiation levels, which suggests that these organisms are capable of coping with such increased mutation frequency.

Despite this general trend, two Chernobyl samples appeared somewhat divergent from the rest, which might be explained by a local heterogeneity effect. One corresponds to Cher4 in the Red Forest, which is subject to the highest doses of radiation measured (25 µSv/h; Table 1), and that, paradoxically, exhibited the largest microbial diversity observed, although showing basically the same taxa as the other biofilm samples. The other sample was Cher8, which systematically exhibited a low diversity both for bacteria and eukaryotes, with typical radiation-resistant Actinobacteria and Ascomycota dominating the sample, and with apparent higher mutation frequencies at the ITS versus the SSU rDNA (Figure 7). However, at single-OTU level, Cher8 showed intermediate mutation rates, which is consistent with its intermediate background radiation exposure (Figures 8 and 9). The diversity pattern of Cher8 might perhaps be related to the presence of increased local metal concentrations within the biofilm. Actinobacteria are important in soils enriched in heavy metals [94], and ascomycete fungi are also known to be highly resistant to important metal concentrations [92], [95]. At any rate, even if these two slightly different samples were excluded from the analyses, the diversity of the remaining samples (including the outgroup Irish sample) is remarkably similar, despite an increase in mutation rate with increasing ionizing radiation.

Our results show that biofilm microbial communities in the most highly irradiated Chernobyl samples are not significantly different from those of non-irradiated samples, despite the occurrence of higher mutation rates. Therefore, the diversity in these biofilms is not necessarily the consequence of ionizing radiation as a predominant selective pressure, although the latter could lead to an increased fine-scale genetic diversity. On the contrary, our results show that characteristic members of these biofilms, such as Actinobacteria, Deinococcales and pigmented ascomycete fungi, are typical formers of biofilms in UV-exposed areas, rocks and monument surfaces or as endolithic organisms. Therefore, their natural adaptations to UV radiation and to desiccation can be considered as pre-adaptations (*ex-aptations*) to cope with the high levels of ionizing radiation occurring at Chernobyl.

## Supporting Information

Figure S1 Accumulation curves for SSU rDNA libraries of biofilm samples in Chernobyl (Cher) and Northern Ireland. In all cases, the number of different sequences observed is plotted against the number of sequences analyzed. The different curves in each plot indicate the various cut-off values considered (grey box) from 0 to 5% divergence. Generally, 97–98% identity is used to define OTU levels representing the species level.
(PDF)

Table S1 Regression and interaction coefficients estimated using the model described in the Methods section.
(PDF)

## References

[1] Moller AP, Mousseau TA (2006). Biological consequences of Chernobyl: 20 years on.. Trends Ecol Evol.

[2] Geras'kin SA, Fesenko SV, Alexakhin RM (2008). Effects of non-human species irradiation after the Chernobyl NPP accident.. Environ Int.

[3] Yablokov AV (2009). 9. Chernobyl's radioactive impact on flora.. Ann N Y Acad Sci.

[4] Zhdanova NN, Redchits TI, Zheltonozhsky VA, Sadovnikov LV, Gerzabek MH (2003). Accumulation of radionuclides from radioactive substrata by some micromycetes.. J Environ Radioact.

[5] Dighton J, Tugay T, Zhdanova N (2008). Fungi and ionizing radiation from radionuclides.. FEMS Microbiol Lett.

[6] Zhdanova NN, Tugay T, Dighton J, Zheltonozhsky V, McDermott P (2004). Ionizing radiation attracts soil fungi.. Mycol Res.

[7] Tugay T, Zhdanova NN, Zheltonozhsky V, Sadovnikov L, Dighton J (2006). The influence of ionizing radiation on spore germination and emergent hyphal growth response reactions of microfungi.. Mycologia.

[8] Kimura S, Ishidou E, Kurita S, Suzuki Y, Shibato J (2006). DNA microarray analyses reveal a post-irradiation differential time-dependent gene expression profile in yeast cells exposed to X-rays and gamma-rays.. Biochem Biophys Res Commun.

[9] Zhdanova NN, Zakharchenko VA, Vember VV, Nakonechnaya LT (2000). Fungi from Chernobyl: mycobiota of the inner regions of the containment structures of the damaged nuclear reactor.. Mycol Res.

[10] Zhdanova NN, Vasilevskaya AI, Artyshkova LV, Sadovnikov YS, Lashko TN (1994). Changes in micromycete communities in soil in response to pollution by long-lived radionuclides emitted in the Chernobyl accident.. Mycol Res.

[11] Dadachova E, Casadevall A (2008). Ionizing radiation: how fungi cope, adapt, and exploit with the help of melanin.. Curr Opin Microbiol.

[12] Dadachova E, Bryan RA, Huang X, Moadel T, Schweitzer AD (2007). Ionizing radiation changes the electronic properties of melanin and enhances the growth of melanized fungi.. PLoS ONE.

[13] Yablokov AV (2009). 11. Chernobyl's radioactive impact on microbial biota.. Ann N Y Acad Sci.

[14] Czirjak GA, Moller AP, Mousseau TA, Heeb P (2010). Microorganisms associated with feathers of barn swallows in radioactively contaminated areas around chernobyl.. Microb Ecol.

[15] Amann RI, Ludwig W, Schleifer KH (1995). Phylogenetic identification and in situ detection of individual microbial cells without cultivation.. Microbiol Rev.

[16] Romanovskaia VA, Rokitko PV, Malashenko Iu R (2000). Unique properties of highly radioresistant bacteria.. Mikrobiologiia Z.

[17] Romanovskaia VA, Rokitko PV, Malashenko Iu R, Krishtab TP, Chernaia NA (1999). Sensitivity of soil bacteria isolated from the alienated zone around the Chernobyl Nuclear Power Plant to various stress factors.. Mikrobiologiia.

[18] Romanovskaia VA, Sokolov IG, Rokitko PV, Chernaia NA (1998). Ecological consequences of radioactive pollution for soil bacteria within the 10-km region around the Chernobyl Atomic Energy Station.. Mikrobiologiia.

[19] Zavilgelsky GB, Abilev SK, Sukhodolets VV, Ahmad SI (1998). Isolation and analysis of UV and radio-resistant bacteria from Chernobyl.. J Photochem Photobiol B.

[20] Charmasson S, Sarradin PM, Le Faouder A, Agarande M, Loyen J (2009). High levels of natural radioactivity in biota from deep-sea hydrothermal vents: a preliminary communication.. J Environ Radioact.

[21] Jolivet E, L'Haridon S, Corre E, Forterre P, Prieur D (2003). *Thermococcus gammatolerans* sp. nov., a hyperthermophilic archaeon from a deep-sea hydrothermal vent that resists ionizing radiation.. Int J Syst Evol Microbiol.

[22] Jolivet E, Corre E, L'Haridon S, Forterre P, Prieur D (2004). *Thermococcus marinus* sp. nov. and *Thermococcus radiotolerans* sp. nov., two hyperthermophilic archaea from deep-sea hydrothermal vents that resist ionizing radiation.. Extremophiles.

[23] Cadet J, Sage E, Douki T (2005). Ultraviolet radiation-mediated damage to cellular DNA.. Mutat Res.

[24] Cadet J, Douki T, Ravanat JL (2010). Oxidatively generated base damage to cellular DNA.. Free Radic Biol Med.

[25] Daly MJ, Gaidamakova EK, Matrosova VY, Vasilenko A, Zhai M (2007). Protein oxidation implicated as the primary determinant of bacterial radioresistance.. PLoS Biol.

[26] Brooks BW, Murray RGE (1981). Nomenclature for *Micrococcus radiodurans* and other radiation-resistant cocci - Deinococcaceae fam. nov. and *Deinococcus* ge.nov., including 5 species.. Int J Syst Bacteriol.

[27] Suzuki K, Collins MD, Iijima E, Komagata K (1988). Chemotaxonomic characterization of a radiotolerant bacterium, *Arthrobacter radiotolerans* – description of *Rubrobacter radiotolerans* gen. nov., comb. nov.. FEMS Microbiol Lett.

[28] de Groot A, Chapon V, Servant P, Christen R, Saux MF (2005). *Deinococcus deserti* sp. nov., a gamma-radiation-tolerant bacterium isolated from the Sahara Desert.. Int J Syst Evol Microbiol.

[29] Chanal A, Chapon V, Benzerara K, Barakat M, Christen R (2006). The desert of Tataouine: an extreme environment that hosts a wide diversity of microorganisms and radiotolerant bacteria.. Environ Microbiol.

[30] Shravage BV, Dayananda KM, Patole MS, Shouche YS (2007). Molecular microbial diversity of a soil sample and detection of ammonia oxidizers from Cape Evans, Mcmurdo Dry Valley, Antarctica.. Microbiol Res.

[31] Wong FKY, Lacap DC, Lau MCY, Aitchison JC, Cowan DA (2010). Hypolithic microbial community of quartz pavement in the high-altitude tundra of Central Tibet.. Microb Ecol.

[32] Ferreira AC, Nobre MF, Moore E, Rainey FA, Battista JR (1999). Characterization and radiation resistance of new isolates of *Rubrobacter radiotolerans* and *Rubrobacter xylanophilus*.. Extremophiles.

[33] Imperi F, Caneva G, Cancellieri L, Ricci MA, Sodo A (2007). The bacterial aetiology of rosy discoloration of ancient wall paintings.. Environ Microbiol.

[34] Rainey FA, Ray K, Ferreira M, Gatz BZ, Nobre MF (2005). Extensive diversity of ionizing-radiation-resistant bacteria recovered from Sonoran Desert soil and description of nine new species of the genus *Deinococcus* obtained from a single soil sample.. Appl Environ Microbiol.

[35] de la Torre JR, Goebel BM, Friedmann EI, Pace NR (2003). Microbial diversity of cryptoendolithic communities from the McMurdo Dry Valleys, Antarctica.. Appl Environ Microbiol.

[36] Pointing SB, Warren-Rhodes KA, Lacap DC, Rhodes KL, McKay CP (2007). Hypolithic community shifts occur as a result of liquid water availability along environmental gradients in China's hot and cold hyperarid deserts.. Environ Microbiol.

[37] Pellerin A, Lacelle D, Fortin D, Clark ID, Lauriol B (2009). Microbial diversity in endostromatolites (cf. fissure calcretes) and in the surrounding permafrost landscape, Haughton impact structure region, Devon Island, Canada.. Astrobiol.

[38] White O, Eisen JA, Heidelberg JF, Hickey EK, Peterson JD (1999). Genome sequence of the radioresistant bacterium *Deinococcus radiodurans* R1.. Science.

[39] Asgarani E, Terato H, Asagoshi K, Shahmohammadi HR, Ohyama Y (2000). Purification and characterization of a novel DNA repair enzyme from the extremely radioresistant bacterium *Rubrobacter radiotolerans*.. J Radiat Res (Tokyo).

[40] Liu Y, Zhou J, Omelchenko MV, Beliaev AS, Venkateswaran A (2003). Transcriptome dynamics of *Deinococcus radiodurans* recovering from ionizing radiation.. Proc Natl Acad Sci U S A.

[41] Slade D, Radman M (2011). Oxidative stress resistance in *Deinococcus radiodurans*.. Microbiol Mol Biol Rev.

[42] Sghaier H, Ghedira K, Benkahla A, Barkallah I (2008). Basal DNA repair machinery is subject to positive selection in ionizing-radiation-resistant bacteria.. BMC Genomics.

[43] Saito T, Terato H, Yamamoto O (1994). Pigments of *Rubrobacter radiotolerans*.. Arch Microbiol.

[44] Tian B, Hua YJ (2010). Carotenoid biosynthesis in extremophilic Deinococcus-Thermus bacteria.. Trends Microbiol.

[45] Fredrickson JK, Li SM, Gaidamakova EK, Matrosova VY, Zhai M (2008). Protein oxidation: key to bacterial desiccation resistance?. ISME J.

[46] Daly MJ (2009). A new perspective on radiation resistance based on *Deinococcus radiodurans*.. Nat Rev Microbiol.

[47] Shestopalov VM (1996). Atlas of Chernobyl exclusion zone.

[48] RPII (2008). Radioactivity monitoring of the Irish Environment.

[49] McAuley IR, Moran D (1989). Radiocesium fallout in Ireland from the Chernobyl accident.. J Radiol Prot.

[50] Yablokov AV, Nesterenko VB, Nesterenko AV (2009). 8. Atmospheric, water, and soil contamination after Chernobyl.. Ann NY Acad Sci.

[51] Schloss PD, Handelsman J (2005). Introducing DOTUR, a computer program for defining operational taxonomic units and estimating species richness.. Appl Environ Microbiol.

[52] Janse I, Bok J, Zwart G (2004). A simple remedy against artifactual double bands in denaturing gradient gel electrophoresis.. J Microbiol Methods.

[53] Sokal R, Michener C (1958). A statistical method for evaluating systematic relationships.. University of Kansas Science Bulletin.

[54] Jaccard P (1901). Distribution de la flore alpine dans le bassin des Dranses et dans quelques régions voisines.. Bulletin de la Société Vaudoise des Sciences Naturelles.

[55] Altschul SF, Madden TL, Schaffer AA, Zhang J, Zhang Z (1997). Gapped BLAST and PSI-BLAST: a new generation of protein database search programs.. Nucleic Acids Res.

[56] Edgar RC (2004). MUSCLE: multiple sequence alignment with high accuracy and high throughput.. Nucleic Acids Res.

[57] Philippe H (1993). MUST, a computer package of Management Utilities for Sequences and Trees.. Nucleic Acids Res.

[58] Jobb G, von Haeseler A, Strimmer K (2004). TREEFINDER: a powerful graphical analysis environment for molecular phylogenetics.. BMC Evol Biol.

[59] Mc Cullagh P, Nelder JA (1989). Generalized linear models.

[60] Team RDC (2011). R: A language and environment for statistical computing.

[61] Fow J (2003). Effects displays in R for generalized linear models.. J Statistical Software.

[62] López-García P, Moreira D (2008). Tracking microbial biodiversity through molecular and genomic ecology.. Res Microbiol.

[63] Cappitelli F, Principi P, Pedrazzani R, Toniolo L, Sorlini C (2007). Bacterial and fungal deterioration of the Milan Cathedral marble treated with protective synthetic resins.. Sci Total Environ.

[64] Pinar G, Ripka K, Weber J, Sterflinger K (2009). The micro-biota of a sub-surface monument the medieval chapel of St. Virgil (Vienna, Austria).. Intl Biodet Biodegr.

[65] Ettenauer J, Sterflinger K, Pinar G (2010). Cultivation and molecular monitoring of halophilic microorganisms inhabiting an extreme environment presented by a salt-attacked monument.. Int J Astrobiol.

[66] Pinar G, Saiz-Jimenez C, Schabereiter-Gurtner C, Blanco-Varela MT, Lubitz W (2001). Archaeal communities in two disparate deteriorated ancient wall paintings: detection, identification and temporal monitoring by denaturing gradient gel electrophoresis.. FEMS Microbiol Ecol.

[67] Pinto FL, Thapper A, Sontheim W, Lindblad P (2009). Analysis of current and alternative phenol based RNA extraction methodologies for cyanobacteria.. BMC Mol Biol.

[68] Madigan MT, Martinko JM, Parker J (2002). Brock Biology of Microorganisms, 10th edition.

[69] Gallego V, Sanchez-Porro C, Garcia MT, Ventosa A (2006). *Roseomonas aquatica* sp. nov., isolated from drinking water.. Int J Syst Evol Microbiol.

[70] Nercessian O, Noyes E, Kalyuzhnaya MG, Lidstrom ME, Chistoserdova L (2005). Bacterial populations active in metabolism of C1 compounds in the sediment of Lake Washington, a freshwater lake.. Appl Environ Microbiol.

[71] Fredrickson JK, Balkwill DL, Drake GR, Romine MF, Ringelberg DB (1995). Aromatic-degrading Sphingomonas isolates from the deep subsurface.. Appl Environ Microbiol.

[72] Hughes KA, Lawley B (2003). A novel Antarctic microbial endolithic community within gypsum crusts.. Environ Microbiol.

[73] Shi T, Fredrickson JK, Balkwill DL (2001). Biodegradation of polycyclic aromatic hydrocarbons by *Sphingomonas* strains isolated from the terrestrial subsurface.. J Ind Microbiol Biotechnol.

[74] Perez-Pantoja D, De la Iglesia R, Pieper DH, Gonzalez B (2008). Metabolic reconstruction of aromatic compounds degradation from the genome of the amazing pollutant-degrading bacterium *Cupriavidus necator* JMP134.. FEMS Microbiol Rev.

[75] Trefault N, De la Iglesia R, Molina AM, Manzano M, Ledger T (2004). Genetic organization of the catabolic plasmid pJP4 from *Ralstonia eutropha* JMP134 (pJP4) reveals mechanisms of adaptation to chloroaromatic pollutants and evolution of specialized chloroaromatic degradation pathways.. Environ Microbiol.

[76] Sait M, Hugenholtz P, Janssen PH (2002). Cultivation of globally distributed soil bacteria from phylogenetic lineages previously only detected in cultivation-independent surveys.. Environ Microbiol.

[77] Kausar J, Ohyama Y, Terato H, Ide H, Yamamoto O (1997). 16S rRNA gene sequence of *Rubrobacter radiotolerans* and its phylogenetic alignment with members of the genus *Arthrobacter*, gram-positive bacteria, and members of the family Deinococcaceae.. Int J Syst Bacteriol.

[78] Barr DJ (1978). Taxonomy and phylogeny of chytrids.. Biosystems.

[79] Tanabe Y, Watanabe MM, Sugiyama J (2005). Evolutionary relationships among basal fungi (Chytridiomycota and Zygomycota): Insights from molecular phylogenetics.. J Gen Appl Microbiol.

[80] Bubrick P, Galun M, Frensdorff A (1984). Observations on free-living *Trebouxia* Depuymaly and *Pseudotrabouxia* Archibald, and evidence that both symbionts from Xanthoria parietina (L) th fr can be found free-living in nature.. New Phytologist.

[81] Sette LD, Passarini MRZ, Rodrigues A, Leal RR, Simioni KCM (2010). Fungal diversity associated with Brazilian energy transmission towers.. Fungal Divers.

[82] Harutyunyan S, Muggia L, Grube M (2008). Black fungi in lichens from seasonally arid habitats.. Stud Mycol.

[83] Sert H, Sumbul H, Sterflinger K (2007). A new species of *Capnobotryella* from monument surfaces.. Mycol Res.

[84] Schabereiter-Gurtner C, Pinar G, Lubitz W, Rolleke S (2001). Analysis of fungal communities on historical church window glass by denaturing gradient gel electrophoresis and phylogenetic 18S rDNA sequence analysis.. J Microbiol Meth.

[85] Garvie LA, Knauth LP, Bungartz F, Klonowski S, Nash TH (2008). Life in extreme environments: survival strategy of the endolithic desert lichen Verrucaria rubrocincta.. Naturwissenschaften.

[86] Nascimbene J, Thus H, Marini L, Nimis PL (2009). Early colonization of stone by freshwater lichens of restored habitats: a case study in northern Italy.. Sci Total Environ.

[87] Berry D, Xi C, Raskin L (2006). Microbial ecology of drinking water distribution systems.. Curr Opin Biotechnol.

[88] Horn M, Wagner M (2004). Bacterial endosymbionts of free-living amoebae.. J Eukaryot Microbiol.

[89] Wong FK, Lacap DC, Lau MC, Aitchison JC, Cowan DA (2010). Hypolithic microbial community of quartz pavement in the high-altitude tundra of central Tibet.. Microb Ecol.

[90] Karsten U, Friedl T, Schumann R, Hoyer K, Lembcke S (2005). Mycosporine-like amino acids and phylogenies in green algae: Prasiola and its relatives from the Trebouxiophyceae (Chlorophyta).. J Phycol.

[91] Karsten U, Lembcke S, Schumann R (2007). The effects of ultraviolet radiation on photosynthetic performance, growth and sunscreen compounds in aeroterrestrial biofilm algae isolated from building facades.. Planta.

[92] Backor M, Fahselt D (2008). Lichen photobionts and metal toxicity.. Symbiosis.

[93] Wornik S, Grube M (2010). Joint dispersal does not imply maintenance of partnerships in lichen symbioses.. Microb Ecol.

[94] Gremion F, Chatzinotas A, Harms H (2003). Comparative 16S rDNA and 16S rRNA sequence analysis indicates that Actinobacteria might be a dominant part of the metabolically active bacteria in heavy metal-contaminated bulk and rhizosphere soil.. Environ Microbiol.

[95] Gadd GM (2007). Geomycology: biogeochemical transformations of rocks, minerals, metals and radionuclides by fungi, bioweathering and bioremediation.. Mycol Res.

