# Supplementary material for: Sunlight-Exposed Biofilm Microbial Communities Are Naturally Resistant to Chernobyl Ionizing-Radiation Levels
Source: PLoS One. 2011 Jul 13;6(7):e21764. doi: 10.1371/journal.pone.0021764 (PMC3135598; doi:10.1371/journal.pone.0021764)
Supplement: Table S1 — Regression and interaction coefficients estimated using the model described in the Methods section. (PDF) [file pone.0021764.s002.pdf]

Table S1. Regression and interaction coefficients estimated using the model described in the Methods section.

**Intercept**	3,091
**16S**	0,041

	**Cher-2**	**Cher-6**	**Cher-8**
**Pop**	-0,188	0,691	-0,504
**16SxPop**	0,035	-0,076	0,041

	**Ref-127**	**Ref-128**	**Ref-2**
**Ref**	-0,061	-0,139	0,201
**16SxRef**	0,008	-0,014	0,006

	**Cher-2**	**Cher-6**	**Cher-8**
**Ref-127**	0,046	-0,077	0,03
**Ref-128**	-0,151	0,052	0,099
**Ref-2**	0,105	0,025	-0,129
