# Supplementary material for: Sunlight-Exposed Biofilm Microbial Communities Are Naturally Resistant to Chernobyl Ionizing-Radiation Levels
Source: PLoS One. 2011 Jul 13;6(7):e21764. doi: 10.1371/journal.pone.0021764 (PMC3135598; doi:10.1371/journal.pone.0021764)
Supplement: Figure S1 — Accumulation curves for SSU rDNA libraries of biofilm samples in Chernobyl (Cher) and Northern Ireland. In all cases, the number of different sequences observed is plotted against the number of sequences analyzed. The different curves in each plot indicate the various cut-off values considered (grey box) from 0 to 5% divergence. Generally, 97–98% identity is used to define OTU levels representing the species level. (PDF) [file pone.0021764.s001.pdf]

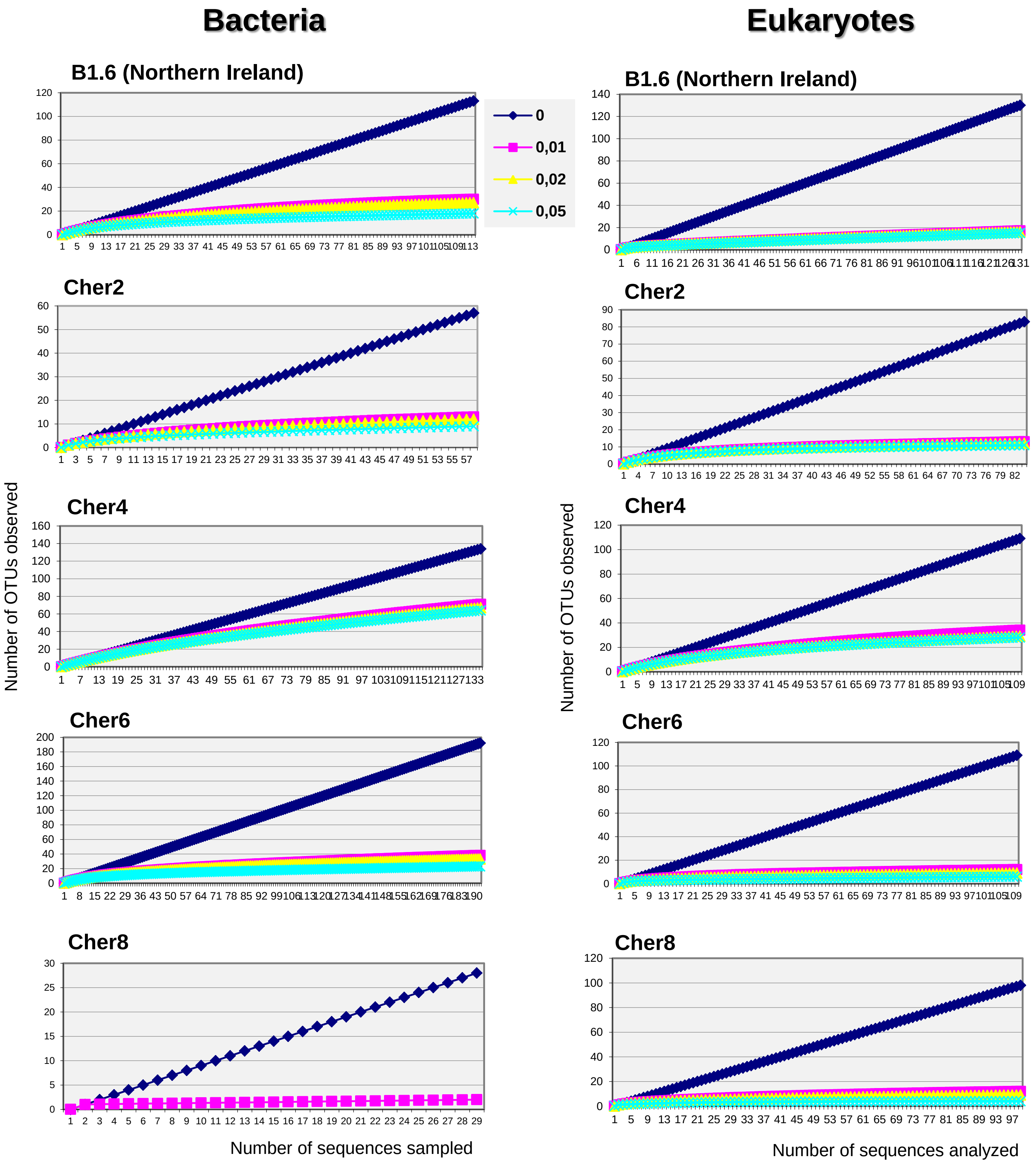

Figure S1. Accumulation curves for SSU rDNA libraries of biofilm samples in Chernobyl (Cher) and North Ireland. In all cases, the number of different sequences observed is plotted against the number of sequences analyzed. The different curves in each plot indicate the various cut-off values considered (grey box) from 0 to 5% divergence. Generally, 97-98% identity are used to define OTU levels representing the species level.
